# Examining Laterality in Masticatory Myofascial Pain

**DOI:** 10.1111/joor.70231

**Published:** 2026-06-05

**Authors:** Rafael Benoliel, Yaron Haviv, Shai Levi, Galit Almoznino, Andra Rettman, Yair Sharav

**Affiliations:** ^1^ Department of Diagnostic Sciences Rutgers School of Dental Medicine Newark New Jersey USA; ^2^ Department of Oral Medicine, Sedation and Imaging Hadassah Medical Center, Hebrew University of Jerusalem, Faculty of Dental Medicine Jerusalem Israel; ^3^ The Institute of Biomedical and Oral Research Hebrew University of Jerusalem, Faculty of Dental Medicine Jerusalem Israel; ^4^ Department of Hematology Tel‐Aviv Sourasky Medical Center Tel‐Aviv Israel

**Keywords:** injuries, masticatory muscles, myalgia, myofascial pain, nausea, temporomandibular disorders

## Abstract

**Objective:**

To characterize unilateral and bilateral masticatory myofascial pain (MMP) in a clinical cohort.

**Materials and Methods:**

Patients meeting criteria for MMP according to the Research Diagnostic Criteria for Temporomandibular Disorders were recruited over a 2‐year period. Demographic and clinical date were recorded. Data for unilateral and bilateral pain were analysed using Mann–Whitney U, Kruskal–Wallis H, and Fisher's Exact tests. Variables showing a *p* < 0.2 were entered into a binary logistic regression model, with pain laterality as the dependent variable.

**Results:**

Patients with unilateral pain exhibited muscle tenderness both ipsilaterally and bilaterally, and patients with bilateral pain likewise demonstrated tenderness bilaterally and unilaterally. Unilateral pain was significantly associated with pain‐related awakening (*p* = 0.03), nausea (*p* = 0.01), and primary pain (*p* = 0.01). These associations were confirmed in the regression analysis. Patients with unilateral and bilateral pain were similar in demographic characteristics.

**Conclusions:**

Unilateral and bilateral masticatory muscle pain differ in three clinical domains. Unilateral pain is significantly associated with pain‐related awakening, primary pain, and nausea. The significance of these findings needs further study in a larger cohort. Additionally, the potential relationship between unilateral/bilateral MMP and headache warrants further investigation.

## Graphical abstract

- Objective: To characterize and compare unilateral and bilateral Masticatory Myofascial Pain (MMP) in a clinical cohort.
- Methods: Patients with MMP according to the RDC‐TMD were recruited over a 2‐year period. Data were analysed with univariable analyses followed by a binary logistic regression model, with pain laterality as the dependent variable.
- Results: Unilateral pain was significantly associated with pain‐related awakening (*p* = 0.03), nausea (*p* = 0.01), and primary pain (*p* = 0.01). These associations were confirmed in the regression analysis. Patients with unilateral and bilateral pain were similar in other demographic characteristics. Reported pain was unilateral or bilateral, but muscle tenderness was present both unilaterally and bilaterally in both groups. Not all tender muscles reproduce clinical pain. Laterality of clinical pain is not always mirrored by underlying muscle tenderness.
- Conclusions: Unilateral pain is significantly associated with: Pain‐related awakening, Primary pain, Nausea. The significance of these findings needs further study in a larger cohort.

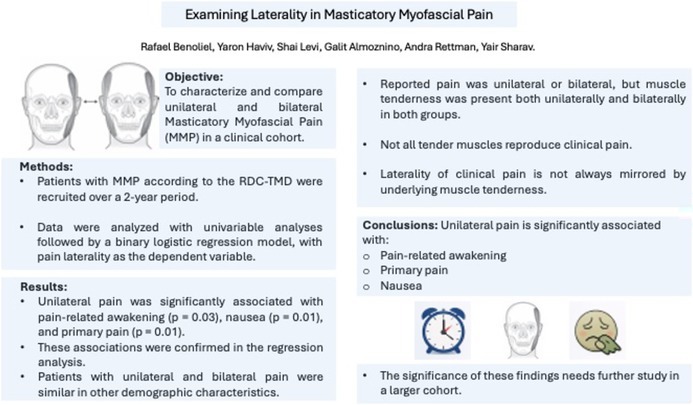

**Abbreviations**
CH: Cluster Headache
DC/TMD: Diagnostic Criteria for Temporomandibular Disorders
ICHD‐3: International Classification of Headache Disorders, version 3
MMP: Masticatory Myofascial Pain
RDC/TMD: Research Diagnostic Criteria for Temporomandibular Disorders
TACs: Trigeminal Autonomic Cephalalgias
TMD: Temporomandibular Disorders
TTH: Tension‐Type Headache
TTS: Total Tenderness Score
VPS: Verbal Pain Scale

## 1 Background

In the diagnosis of orofacial pain and headache the laterality of pain is often a crucial factor. Certain disorders such as trigeminal neuralgia and cluster headache are exclusively, or overwhelmingly, unilateral [1, 2, 3]. In contrast tension type headache (TTH) is predominantly bilateral [3]. Other disorders such as migraine are usually unilateral but may present bilaterally in a substantial proportion of patients [4].

Masticatory myofascial pain (MMP) has been largely considered a unilateral disorder [5, 6, 7], although it has increasingly been recognized that bilateral presentations may also occur [5]. Regarding muscle tenderness, an early study on MMP patients reported both unilateral and bilateral tenderness in the masseter, temporalis, medial pterygoid, and cervical muscles [8]. In contrast, a more recent study on female MMP patients exclusively reported bilateral signs [9], with one side being the more symptomatic.

However, classification criteria for MMP do not specify whether the pain is unilateral or bilateral nor do they refer to unilateral versus bilateral muscle tenderness [10, 11]. Laterality of pain and associated muscle tenderness may be important. Possibly unilateral and bilateral MMP may be different pathophysiologic, clinical, and therapeutic entities.

Human experiments shed some light on muscle pain. Intramuscular injection of algesic substances induces pain and referral patterns characteristic of craniofacial myofascial conditions. Injections into the sternocleidomastoid and trapezius muscles produce localized pain with referral to regions associated with TTH location [12, 13, 14, 15]. In contrast injection into the masseter, medial or lateral pterygoids refers pain to the teeth, angle of the mandible and temporomandibular joint and resembles the pain pattern observed in MMP as defined in the DC TMD criteria [13, 14]. Quality and intensity of resultant pain do not differ significantly between injection sites. These experimental findings would suggest a prominent role for muscle nociceptors in the location and referral patterns of reported pain in TTH and MMP. Pain referral patterns also involve central convergence of peripheral afferents onto second order neurons in subnucleus caudalis, central sensitization with expansion of receptive fields [16] and activation of convergent thalamic neurons [17]. Bilateral muscle pain has been induced experimentally. Investigators applied five pain‐inducing methods to volunteers but did not consistently produce masticatory muscle pain in subjects [18]. Certain individuals appeared to be very susceptible to developing pain during or after most of the exercises. These susceptible individuals demonstrated a bilateral muscle pain pattern after the unilaterally stressful exercises [18]. This would suggest that clinical bilaterality is associated with individual predisposition, possibly a tendency to central sensitization and/or a disorder of pain modulation.

With scarce literature on the subject, we undertook this study to better understand the clinical features of bilateral versus unilateral MMP. Our working hypothesis was that there would be significant differences in the clinical features and demographic characteristics.

## 2 Materials and Methods

We collected patients attending our pain clinic with a complaint of orofacial pain which was associated with a diagnosis of MMP [19]. Demographic data was collected and recorded. A detailed pain history was recorded including location, quality, severity, and associated features. We recorded the facial pain's temporal pattern (time from onset, attack duration, frequency). All patients rated their pain at baseline employing a verbal pain scale (VPS) of 0 (no pain) to 10 (maximal pain imaginable). Location of the orofacial pain, including the laterality, was recorded verbally and on a face diagram. Pain quality was assessed by asking patients to choose one or more of the following descriptive terms: electrical, stabbing, throbbing, pressure, burning, annoying or any combination. These arbitrary terms are in routine use in our clinic to provide rapid assessment of pain quality. Patients were asked if the pain specifically wakes them from sleep. Answers were carefully assessed to ensure that the patient was reporting awakening specifically related to pain, and not for example random awakenings (to drink water, for micturition). We recorded if there were any associated nausea or other systemic symptoms, body pain, dizziness or cranial autonomic symptoms. We termed patients with pain onset following a clear traumatic event as ‘post‐traumatic’ relative to a ‘primary’ onset in the rest of the patients. Trauma was divided into two subgroups: macrotrauma (road traffic accidents and altercations) and dental (surgical or other interventions lasting more than 1 h).

Clinical examination included a routine physical of the head and neck, the masticatory apparatus, the dental and periodontal tissues, and an examination of the cranial nerves. The masticatory apparatus (temporomandibular joint, masticatory muscles) was examined for sensitivity to palpation. The cervical muscles were also examined. Muscles were palpated with a slow rotatory movement and held for 5 s with a precalibrated pressure of ~1 kg [10]. Muscles examined included masseter, temporalis, medial pterygoid, sternocleidomastoid, trapezius and the suboccipital group (as one) bilaterally [20, 21]. Pain/tenderness on muscle palpation needed to replicate ongoing pain. Muscle tenderness was graded on a 4‐point Likert scale from 0 to 3 (0 = none, 1 = mild, 2 = moderate, 3 = severe). The scores are summed to provide a total tenderness score (TTS) [20, 21]. Imaging is requested as needed. The above assessment is performed at baseline when patients first arrive for treatment, and before medications were prescribed. These patients were collected at the Orofacial Pain Clinic, Faculty of Dentistry, Hadassah‐The Hebrew University, Jerusalem as part of a time‐limited, 2‐year (2005–2007) prospective study on various aspects of orofacial pain. A diagnosis of MMP was established according to the Research Diagnostic Criteria for Temporomandibular Disorders [19] which were relevant at the time of the study. Additional inclusion criteria were that pain had to be present for 3 months or more, and the patient was able to independently communicate. Exclusion criteria were comorbid arthralgia of the temporomandibular joint, and current ingestion of central or peripheral analgesic agents. The Hadassah Medical Center Helsinki committee (HMO‐0473‐14) approved this study which complied with accepted ethical standards.

All patients verbally consented to participate in the study. The data that support the findings of this study are available on request from the corresponding author. The data are not publicly available due to privacy or ethical restrictions.

### 2.1 Statistical Analyses

Data was tabulated and analysed with SPSS (version 31 for Macintosh), with two‐tailed alpha for significance set at 0.05. The data was not normally distributed (all applied Kolmogorov–Smirnov tests *p* < 0.05) and therefore nonparametric analyses were performed.

Differences in continuous variables between unilateral and bilateral cases were analysed with the Mann–Whitney U test for two categories, the Kruskal Wallis H test for > 2 categories, and associations between nominal variables with a Fisher's exact test (F). Analysed continuous data are reported as medians with interquartile ranges (IQR), otherwise as mean ± standard deviation.

A backward stepwise regression analysis was used to analyze multivariable correlation on laterality of pain. Independent variables were selected for the model, based on a *p* < 0.2 cut‐off in the univariable analyses.

## 3 Results

### 3.1 Univariable Analyses

#### 3.1.1 Total Cohort (Tables 1 & 2)

**TABLE 1:** Characterization of laterality of facial pain by demographic variables.

Variable		Laterality		Total	OR	Stats *(p)*
		Unilateral	Bilateral			
Sex *n* (%)	Males	26 (36%)	16 (34%)	42	1.82 (0.75–4.40)	0.21*
	Females	47 (64%)	31 (66%)	78		
Age of Onset (y)	Median (IQR)	31 (23–45)	37 (26–55)	—	1.004 (0.998–1.009)	0.22**
Time to first visit (m)	Median (IQR)	8 (3–30)	6 (3–30)	—	0.999 (0.998–1.001)	0.69**
Pain Frequency (d/m)	Median (IQR)	28 (28–28)	28 (28–28)	—	0.998 (0.985–1.010)	0.64**
Pain Duration (h)	Median (IQR)	24 (3–24)	15 (3–24)	—	0.999 (0.996–1.002)	0.95**
Primary/Secondary *n* (%)	Primary	45 (62%)	18 (38%)	63	2.59 (1.22–5.50)	**0.01** *
	Secondary	28 (38%)	29 (62%)	57		

*Note:* Bold values are statistical results that are significant.

Abbreviations: d, days; h, hours; IQR, Inter Quartile Range; m, months; *n*, sample size; OR, Odds Ratio 95% Confidence Intervals; y, years.

* Fisher's Exact test.

** Mann–Whitney U test.

**TABLE 2:** Characterization of laterality of facial pain by clinical variables.

Variable		Laterality		Total	OR	Stats *(p)*
		Unilateral	Bilateral			
Muscle pain *n* (%)	Unilateral	25 (34%)	21 (45%)	46	1.55 (0.73–3.29)	0.34*
	Bilateral	21 (66%)	26 (55%)	47		
Nausea *n* (%)	Yes	12 (16%)	1 (2%)	13	9.01 (1.14–71.42)	**0.01** *
	No	61 (84%)	46 (98%)	107		
Dizziness *n* (%)	Yes	24 (33%)	16 (34%)	40	1.1 (0.49–2.29)	1.00*
	No	49 (67%)	31 (66%)	80		
TTS	Median (IQR)	6 (4–9)	6 (4–10)	—	1.012 (0.989–1.036)	0.57**
VPS (0–10)	Median (IQR)	7 (6–8.5)	8 (6–8)	—	0.998 (0.944–1.034)	0.49**
Awaken *n* (%)	Yes	22 (30%)	6 (13%)	28	2.95 (1.09–7.94)	**0.03** *
	No	51 (70%)	41 (87%)	92		
IO (mm)	Median (IQR)	41.5 (37–46)	45 (39.5–48.5)	—	1.007 (0.995–1.019)	0.15**

*Note:* Bold values are statistical results that are significant.

Abbreviations: IO, interincisal opening; IQR, Inter Quartile Range; *n*, sample size; OR, Odds Ratio 95% Confidence Intervals; TTS, total tenderness score; VPS, verbal pain score.

* Fisher's Exact test.

** Mann–Whitney U test.

The total cohort was made up of 120 patients (31 Males, 26%; 89 Females, 74%) with 61% (*n* = 73) reporting unilateral pain and 39% (*n* = 47) reporting bilateral pain. Mean onset age was 37.6 ± 16.8 years. Pain occurred daily in all patients, while 77 (64%) patients reported continuous pain, 43 (36%) with well‐defined attacks of pain lasting 1–12 h (median 3 h, IQR = 1–4).

In the total group the distribution of the VPS for patients reporting pain‐related awakening was significantly different to those with no awakening (U = 775.5, df = 1, *p* = 0.04). In patients reporting pain‐related awakening the median VPS was 8 (IQR 6.5–10) and in those not reporting awakening 7 (IQR 6–8). Similarly, the distribution of TTS data was significantly different between those awakened by pain and those not (U = 933.5, df = 1, *p* = 0.03). The median TTS in patients reporting pain‐related awakening was 7.5 (IQR 6–11) and in those not reporting awakening 6 (IQR 4–8).

When examining primary versus post traumatic pain in this larger cohort, there were no significant differences between these in the distribution of values for VPS, reports of awakening, total tenderness score, and the presence of nausea. Further analysis of dental trauma, macrotrauma, and primary pain with a Kruskal‐Wallis test revealed no significant differences in VPS and total tenderness scores. No differences between the three groups were found in rates of pain‐related awakening, dizziness, and nausea.

### 3.2 Examining Laterality

The distributions of sex, age of onset, time to first visit, VPS, interincisal opening, TTS, pain frequency, and pain attack duration were not significantly different across patients complaining of unilateral versus bilateral pain, see Tables 1 and 2. There were no reports of cranial autonomic symptoms or of associated body pain.

In the patients with an associated history of trauma 12 (21%) reported dental treatment and 45 (79%) macrotrauma. An association between trauma and the onset of pain was recorded in 28 (38%) of patients with unilateral pain and 29 (62%) of patients with bilateral pain. Primary pain, not associated with a history of trauma or other event, was reported by 45 (62%) of unilateral cases and 18 (38%) of bilateral cases. This distribution was significantly different (F, *p* = 0.01).

Patients with unilateral pain reported both unilateral (*n* = 25, 34%) and bilateral (*n* = 21, 66%) muscle tenderness, see Table 2. Those with bilateral pain also reported both unilateral (*n* = 21, 45%) and bilateral (*n* = 26, 55%) muscle tenderness. This distribution was not statistically significant (F, *p* > 0.05), see Table 2.

No cases of phono/photo‐phobia or vomiting were reported. Nausea was observed in 16.4% (*n* = 12) of unilateral pain cases and 2.1% (*n* = 1) of bilateral cases. This distribution was significantly different (F, *p* = 0.01). Dizziness associated with pain was reported by 32.9% (*n* = 24) of unilateral cases and 34% (*n* = 16) of bilateral cases and was not significant (F, *p* > 0.05).

Awakening due to pain was reported by 30% (*n* = 22) of patients with unilateral pain and 12.8% (*n* = 6) of patients with bilateral pain. These distributions were significantly different (F, *p* = 0.03).

### 3.3 Multivariable Analysis

The final model in the logistic regression included pain related awakening (*p* = 0.01), whether the pain was primary or secondary (*p* = 0.01), and the presence of nausea (*p* = 0.04), Table 3. Pain that awakened the patient had a 3.8 (95% CI 1.3–10.7) times likelihood of having unilateral pain. Primary pain was associated with a 2.8 (95% CI 1.2–6.2) times likelihood of having unilateral pain. Finally, nausea was associated with an 8.8 (95% CI 1.1–71) times likelihood of having unilateral pain. The Hosmer and Lemeshow Test for goodness of fit was *p* = 0.4 (χ^2^ = 3, df = 3) indicating a good fit for the regression analysis. Collinearity statistics were within acceptable limits; see Table 3.

**TABLE 3:** Significant associations with unilateral pain on univariable and multivariable analyses. Collinearity statistics.

Parameter	Univariable analysis^a^		Multivariable logistic regression analysis		Collinearity statistics	
	df	*p*	*p*	OR (95% C.I.)	Tolerance	VIF
Nausea	1	0.01	0.04	8.8 (1.1–71)	0.984	1.017
Primary pain	1	0.01	0.01	2.8 (1.2–6.2)	0.977	1.024
Awakens	1	0.03	0.01	3.8 (1.3–10.7)	0.986	1.014

^a^ Fisher's Exact test.

## 4 Discussion

This study examined the differences between unilateral and bilateral MMP. While reported pain was either unilateral or bilateral, underlying muscle tenderness was present both unilaterally and bilaterally in both groups. Unilateral MMP had bilateral muscle tenderness (muscle pain on palpation will be referred to as “tenderness”) more often. This raises some questions regarding the relationship between reported pain and muscle tenderness. Clearly, not all tender muscles reproduce clinical pain, and clinical pain is not always mirrored by underlying muscle tenderness.

### 4.1 Muscle Tenderness

The presence of muscle tenderness in the role of pain has been questioned in the headache field and related specifically to TTH. It is unclear whether the presence of pericranial muscle tenderness is the cause or the result of the pain. Many TTH patients display increased muscle tenderness, and this is considered important in its pathophysiology [3, 22]. Increased pericranial tenderness is the most significant abnormal finding in patients with TTH. It is typically present interictally, is exacerbated during actual headache and increases with the intensity and frequency of headaches. Increased tenderness is very probably of pathophysiological importance. However, patients with TTH may present without pericranial muscle tenderness so that the ICHD 3 classifies TTH as with or without pericranial tenderness. Additionally, other mechanisms may underlie muscle tenderness. Migraine patients typically have tenderness of masticatory and cervical muscles [23]. Interestingly, one study found muscle tenderness on both the reported painful and nonpainful sides in patients with migraine, indicating bilateral muscle sensitivity regardless of headache location [24]. Muscle tenderness was more marked on the symptomatic side.

It is generally found that central sensitization is present in MMP patients [25]. Although speculative, it is possible that in our series the presence of central sensitization is a factor. This would be expressed in the patients with bilateral pain related to unilateral muscle tenderness and in patients with unilateral pain and bilateral muscle tenderness. Future studies should examine parameters associated with central sensitization, such as temporal summation and conditioned pain modulation.

### 4.2 MMP and Associated Pain Comorbidity

Bilateral muscle pain is often associated with fibromyalgia [26, 27, 28]. MMP pain intensity is increased when comorbidities such as fibromyalgia and lower back pain are present but this was not seen for headaches [29]. Indeed, MMP patients who present with widespread pain seem to respond to therapy differently [30]. There are indications that these comorbidities and MMP share a basic disorder in pain modulation and psychosocial factors. None of our patients reported comorbid body pain, and the intensity of pain was not different between unilateral versus bilateral pain. Why some patients present with bilateral pain remains elusive. Possibly these may represent a progression from unilateral to bilateral pain in MMP. However, the onset period was no different between the unilateral and bilateral group suggesting that bilaterality may present de novo.

Interestingly, episodic TTH in migraine sufferers responds to sumatriptan, a migraine specific drug, whilst in non‐migraine patients it does not [31]. This may suggest that mild migraines may phenotypically be very similar to episodic TTH. The presence of nausea is usually associated with migraine and at times with the TACs. On average 50% of migraineurs vomit during an attack and 80% report nausea [32, 33]. These are also common in cluster headache attacks affecting around a quarter of patients [34]. Although the unilateral cases were largely primary, woke from sleep and had higher rates of nausea (unilateral 16.4% vs. bilateral 2%), none of our unilateral cases reported comorbid migraine or TAC and there were no reports of cranial autonomic symptoms. An interesting case was recently reported [35] where a temporomandibular disorder masqueraded as a migraine suggesting overlapping symptomatology. This discussion is speculative as we don't have any patients with comorbid headache; nevertheless, this possible association should be investigated in a larger sample.

Neurovascular headaches such as migraine are largely unilateral. Migraine headache tends to become bilateral with chronicity, and factors associated with progression of episodic to chronic migraine include increased frequency of episodic migraine, female sex, lower socioeconomic status, and being unmarried. Other factors include comorbid pain syndromes and previous neck or head injury [36]. No comorbid pain was seen in our patients, and as in migraine, bilateral pain was significantly related to a history of trauma. This suggests similar responses to injury in MMP and migraine patients.

As stated, bilateral MMP may be related to TTH. Both the presentation and the distribution of muscle tenderness are similar. The major difference between TTH and MMP is reflected in the laterality and the involved muscles, which are mainly cervical [13, 14]. Possibly the pathophysiology of MMP shares mechanisms with entities such as regional myofascial pain, tension‐type headache, and fibromyalgia [37]. These may all form a spectrum of clinical presentations of the same disorder. Of relevance, MMP has been significantly associated with a number of comorbidities such as fibromyalgia [26, 27], migraine [38, 39, 40], and tension‐type headache [41, 42].

### 4.3 Pain Related Awakening

Musculoskeletal pain and disturbed sleep often create a bidirectional cycle, where pain causes poor sleep, and poor sleep reduces pain tolerance, exacerbating discomfort. This interaction affects 50%–88% of chronic pain patients, leading to severe insomnia, poor daytime functioning, and heightened pain sensitivity [43]. This high rate of pain‐sleep interaction may explain the level of pain related awakening observed in this study.

Awakening was significantly more frequent in unilateral pain. The mechanisms underlying this difference are unclear. Usually, awakening in facial and head pain is associated with Migraines [44] or TACs [45] and forms part of the sleep‐pain mechanisms rather than being solely related to pain severity. These are both largely unilateral disorders, and it is interesting to speculate if unilateral muscle pain may have any relation to a mild orofacial migraine.

Although research indicates that up to 40% of individuals with TTH experience morning headaches [46], patients are not usually woken from sleep by TTH. In muscle pain patients, awakening is usually associated with pain severity and in our overall group this held true. Indeed, when looking at the whole cohort, which expands our number of subjects and improves statistical power, there was a significant association between waking and increased VPS. In our study, patients with unilateral pain were awakened significantly more frequently (30%) compared to patients with bilateral pain (12.8%). However, there was no difference in distribution of VPS between unilateral and bilateral pain cohorts, suggesting that a further mechanism may be involved in pain‐related awakenings. In the total cohort, we found significant differences between unilateral and bilateral cases in the total tenderness score which may contribute to pain‐related awakening. Possibly, movement during sleep may cause more pain in patients with high muscle tenderness, adding a further explanation to the phenomenon of awakening from sleep.

### 4.4 Trauma and MMP

Trauma, whether as dental or macrotrauma, was significantly associated with bilateral pain. The appearance of MMP following trauma is well documented [47, 48] and a history of trauma is present in a significant number of patients with MMP [47, 49, 50, 51, 52]. Dental surgery has also been found to increase the prevalence and symptomatology of TMD [53, 54]. The exact mechanisms of how trauma leads to MMP are unclear but may include direct/invasive muscle damage, stretch injuries to muscle or prolonged immobilization associated with the reduction of fractured jaws. Theoretically extensive injuries (macrotrauma) to the face may induce bilateral pain more often than microtrauma. Our sample size was too small to address this issue. Indirect injury of brain tissue also may lead to persistent head and face pain, although there is no correlation between the degree of injury and the incidence or severity of the pain. Shear forces applied to the brain may result in severe damage. Following even relatively minor head trauma, progressive and extensive axonal injury can occur, commonly known as diffuse axonal injury [55, 56]. Whether post‐traumatic MMP patients suffer more severe symptoms, or are more resistant to treatment, is unclear [57, 58, 59]. In our patient series we found no differences in VPS, pain‐related awakening, or TTS in primary versus posttraumatic MMP.

## 5 Conclusions

This study demonstrates that unilateral and bilateral MMP differ across three distinct clinical domains. Unilateral pain is significantly associated with pain‐related awakening from sleep, nausea, and a primary (non‐traumatic) onset, whereas bilateral pain is more frequently associated with a history of trauma. Despite differences in reported pain laterality, muscle tenderness was frequently bilateral in both groups, underscoring a dissociation between pain location and underlying muscle sensitivity. These findings suggest that unilateral and bilateral MMP may represent partially distinct clinical phenotypes, potentially involving different pathophysiological mechanisms. The associations of unilateral pain with nocturnal awakening and nausea raise the possibility of an overlap with neurovascular headache‐related mechanisms, warranting further investigation.

## 6 Limitations

Our sample size is small and replication of these findings in larger cohorts, with the inclusion of measures of central sensitization and pain modulation, is needed to clarify the clinical and mechanistic significance of the laterality reported pain and muscle tenderness in MMP.

Our discussion is purposely speculative to point out important gaps in our knowledge on laterality of pain and muscle tenderness in MMP.

## Author Contributions

**Shai Levi:** data curation, formal analysis, writing – review and editing. **Yaron Haviv:** conceptualization, data curation, writing – review and editing, methodology. **Andra Rettman:** data curation, writing – review and editing. **Galit Almoznino:** data curation, formal analysis, writing – review and editing. **Rafael Benoliel:** conceptualization, data curation, supervision, formal analysis, writing – review and editing, writing – original draft, methodology. **Yair Sharav:** conceptualization, data curation, supervision, writing – review and editing, writing – original draft, methodology.

## Conflicts of Interest

Rafael Benoliel receives remuneration as editor in chief for the Journal of Oral Facial Pain and Headache. All other authors declare no conflicts of interest.

## Data Availability

The data that support the findings of this study are available on request from the corresponding author. The data are not publicly available due to privacy or ethical restrictions.
